# Prevalence and associated factors of contraceptive discontinuation among reproductive-age women in Ethiopia: using 2016 Nationwide Survey Data

**DOI:** 10.1186/s12978-020-01032-4

**Published:** 2020-11-07

**Authors:** Birye Dessalegn Mekonnen, Chalachew Adugna Wubneh

**Affiliations:** 1 Department of Nursing, Teda Health Science College, P.O.BOX: 790, Gondar, Ethiopia; 2 Department of Pediatric and Child Health Nursing, School of Nursing, College of Medicine and Health Science, University of Gondar, P.O.BOX: 196, Gondar, Ethiopia

**Keywords:** Prevalence, Contraceptive, Discontinuation, Factors, Women, EDHS 2016, Ethiopia

## Abstract

**Background:**

Contraceptive discontinuation for reasons other than the desire for pregnancy is associated with mistimed and unwanted pregnancies, unwanted births and unsafe abortions which has increased risks of maternal morbidity and mortality. However, research on the identification of factors that are associated with contraceptive discontinuation in Ethiopia is limited. Therefore, this study aimed to determine the prevalence of contraceptive discontinuation and associated factors among reproductive-age women in Ethiopia, using recent national survey data.

**Methods:**

A population-based cross-sectional study was conducted using secondary data analysis from of 2016, Ethiopian Demographic Health Survey. A total of 10,871 reproductive-age women were included. The analysis was performed using SPSS version 20 statistical package. Bivariate and multivariate logistic regression analysis was conducted to examine significant factors of contraceptive discontinuation, and statistical significance was declared at p-value < 0.05.

**Results:**

The prevalence of discontinuation for all contraceptives methods among reproductive-age women was 32.2% (95% C.I 31.2, 33.1). Rural residence (AOR = 1.94, 95% C.I 1.65, 2.28), women with no formal education (AOR = 1.68, 95% C.I 1.30, 2.17), women having no children (AOR = 1.95, 95% C.I 1.19, 3.58), husband desire for children (AOR = 2.57, 95% C.I 2.03, 3.26), women self-decision when using a contraceptive (AOR = 0.54, 95% C.I 0.38, 0.77), joint decision when using a contraceptive (AOR = 0.38, 95% C.I 0.29, 0.48), didn’t discuss about FP with healthcare worker (AOR = 1.28, 95% C.I 1.06, 1.54) and didn’t get information about side effects (AOR = 2.01, 95% C.I 1.59, 2.52) were factors significantly associated with contraceptives discontinuation.

**Conclusion:**

The prevalence of contraceptive discontinuation among reproductive-age women was high and multiple factors determined it. Thus, counseling on side effects, availability of other contraceptive methods, and partner involvement in decision-making process by health care providers are strongly recommended. In addition, women empowerment should be promoted so that women are able to liberally decide on when and how many children they wish to have.

## Plain language summary

High proportion of contraceptive discontinuation is a public health concern that frequently associated with unintended pregnancies, unwanted births, and unsafe abortions which have increased risks of pregnancy and childbirth-related maternal morbidity. The decision to continue or discontinue the use of contraceptives could have influenced with a number of factors. A greater understanding of these factors will inform policymakers, programmers, and other stakeholders to strengthen family planning and other health intervention programs to achieve the SDGs targeted maternal and under-5 child mortality reduction. Thus, the main objective of this study was to assess the prevalence of contraceptive discontinuation and associated factors among reproductive-age women in Ethiopia, using EDHS 2016 dataset. A population-based cross-sectional study design was conducted using secondary data analysis from the EDHS 2016. The analysis was done from a total of 10,871 reproductive age women interviewed in the survey. For all women age 15–49 who started an episode of contraceptive use preceding the 2016 EDHS, 32.2% of the episodes were discontinued within 12 months. The finding has an implication for policymakers, programmers, health care providers, and other stakeholders to evaluate and strengthen the accessibility of different contraceptive methods and family planning service quality. The findings of this study strongly recommended the provision of quality counseling on side effects and availability of other contraceptive methods, women empowerment, and partner involvement in decision-making process regarding contraceptive use.

## Introduction

Family planning (FP) is vital to achieve maternal and child health-related Sustainable Development Goals (SDG), and which can contribute to the reduction of more than two-fifth of maternal mortality and about one-fifth of deaths in under-5 children [1, 2]. Though consistent use of contraception is important in the reduction of unintended pregnancies and abortions, many women do not utilize contraceptive methods or not using contraceptives consistently [3, 4]. Despite, women’s wish to limit or space births, contraceptive discontinuation suggest an important reproductive health problem [5, 6].

Contraceptive discontinuation is a public health concern that has a negative effect on women’s reproductive health outcomes [7, 8]. A high proportion of contraceptive discontinuation without the desire of women to get pregnant is associated with unintended pregnancies, unwanted births and unsafe abortions which have increased risks of pregnancy and childbirth related maternal morbidity and poor infant and child health outcomes [8–10].

Contraceptive discontinuation can detrimentally affect the effect of FP programs and has implications for demographic growth. An analysis of Demographic and Health Surveys (DHS) conducted in 34 countries estimated that among women who had ever used a modern method, about 38% discontinued using a modern method of women in spite of the continuing need for family planning; and past use accounted for at least 50% of all women with unmet need in 16 of the countries [11]. Moreover, evidence indicated that unmet need was reported as high where contraceptive discontinuation is high and contraceptive prevalence is low [12, 13]. On the other hand, an analysis of DHS datasets in 14 countries revealed that a substantial number of women had not switched to another contraceptive method; stayed at risk for pregnancy [14].

In Ethiopia, the use of any contraceptive methods among reproductive-age women has increased. Despite these increases, the Ethiopia demographic and health survey (EDHS) 2016 reported that many women (35%) discontinued their contraceptive method with only 6% of the women switched to another method [15].

The decision to continue or discontinue the use of contraceptives could be influenced by a number of factors including; age of woman, number of living children (parity), desired number of children, having television and radios, decision-maker to use contraceptive, husband/partner support, perceived benefit to the FP, perceived contraceptive harm, duration of contraceptive use, counseling on FP and contraceptive side effects, experience of side effects, access and availability different type of contraceptive [10, 14, 16–19].

There have been a number of studies into the identification of factors associated with contraceptive use in Ethiopia, but few on the identification of factors associated with contraceptive discontinuation. A greater understanding of these issues will inform policymakers, programmers and other stakeholder to strengthen family planning and other health intervention programs to achieve the SDGs targeted maternal and under-5 child mortality reduction. To address this gap, a nationwide analysis from the recent EDHS 2016 was conducted. Thus, the main objective of this study was to assess the prevalence of contraceptive discontinuation and associated factors among reproductive-age women in Ethiopia, using EDHS 2016 dataset.

## Methods

### Study design and source of data

A population-based cross-sectional study design was conducted using secondary data analysis from the EDHS 2016. The EDHS was carried out from January 18, 2016 to June 27, 2016. The data management and cleaning process was carried out from May 11 to 23, 2020. A two-stage stratified sample design was utilized in this survey. At first, overall 645 enumeration clusters were randomly selected proportional to the household size; of which 202 were in urban and 443 were in rural. The second stage involved with an equal probability systematic selection of 28 households per cluster from the newly formed household list [15].

In the Ethiopian DHS 2016, nationally representative of 18,008 households were selected. Of these, 16,650 households were identified and interviewed yielding a response rate of 92.5%. From 16,650 interviewed households, 16, 583 were identified as eligible women; and a total of 15,683 reproductive-age women were interviewed yielding a response rate of 94.6% [15]. The data used in this analysis were weighted to adjust for non-response and variations in probability of selection. Furthermore, the data used was limited to responses from women who had ever used a method of both modern and traditional contraception in the 12 months prior to the survey, and where full contraceptive histories were provided. Thus, the analysis was restricted to 10,871 (weighted sample) reproductive-age women. The data for contraceptive discontinuation and associated factors were taken from a woman’s questionnaire.

### Study variables

#### Dependent variable

The outcome variable for this study was contraceptive discontinuation (yes/no) among reproductive-age women (women aged 15–49 years), which is measured as the percentage of reproductive-age women who used a method of contraception in the last 12 months prior to the survey, but were not using a method at the time of data collection.

#### Explanatory (independent) variables

Age, religion, marital status, educational level, residence, current working status, wealth status, number of live children, husband fertility desire, women fertility preference, decision on contraceptive use, knowledge about contraceptives, history of visiting health facility, discussion with healthcare provider on FP, information regarding availability of other contraceptives and counseling about side effects of contraceptives.

### Statistical analysis

The data for this analysis were extracted from EDHS 2016 and accessed from the MEASURE DHS database at https://www.dhsprogram.com/data/dataset_admin/login_main.cfm. The data sets were downloaded in SPSS format with permission from MEASURE DHS. To produce the proper representation of FP information and related factors as well as to adjust for differences in the probability of selection and interview between cases in a sample due to design, coincidence, or corrections for differential response rates, sampling weight was applied to an individual interview unit. The data analysis was done using SPSS 20 statistical software packages. Descriptive statistics were used to summarize the distribution of selected background characteristics of women. Bivariate and multivariate logistic regression model was performed to assess the association between predictor variables and the outcome variable of the study. Those determinant variables with p ≤ 0.2 in the bivariate logistic analysis were included in the multivariate logistic regression analysis. Adjusted odds ratio (AOR) with 95% confidence interval (CI) was calculated to predict the strength of association between factors and contraceptive discontinuation. The variance inflation factor (VIF) was used to check multicollinearity between covariates and the goodness of fit was checked using the likelihood ratio test. Finally, a p-value of less than 0.05 in the multivariate analysis was considered statistical significance.

## Results

### Socio-demographic and economic characteristics of participants

Out of the 10,871 reproductive age women included in this study, 8101 (74.5%) were married and 7800 (71.8%) were from rural areas. The mean age of participants was 26.4 years (SD ± 8.1), where more than one fourth 3090 (28.4%) of participants were in the age group between 15 and 19 years. Nearly two-third, 7096 (65.3%) were Orthodox by religion followed by 3238 (29.8%) of Muslim followers. More than two-fifth, 4664 (42.9%) of the respondents have no formal education and only 3717 (34.2%) have primary education. Out of the total study women, 3189 (29.3%) were categorized as poorest in wealth status whereas, only 1781 (16.4%) were categorized as richest. Regarding regional distributions, the largest percentage of respondents were from the Southern Nations, Nationalities, and Peoples’ Region (SNNPR) (12.1%) and Oromia region (12.1%) (Table 1).

**Table 1:** Socio-demographic characteristics of women who ever used a contraceptive method within 5 years before the 2016 EDHS (weighted)

Variables	Number	Percent
Age		
15–19	3090	28.4
20–24	1743	16.1
25–29	2032	18.7
30–34	1830	16.8
35–39	1468	13.5
40–44	483	4.4
45–49	225	2.1
Marital status		
Married	8101	74.5
Never married/divorced/widowed/separated	2770	25.5
Religion		
Orthodox	7096	65.3
Muslim	3238	29.8
Protestant	393	3.6
Catholic	144	1.3
Place of residence		
Urban	3071	28.2
Rural	7800	71.8
Educational level		
No formal education	4664	42.9
Primary school	3717	34.2
Secondary school	1613	14.8
Higher education	877	8.1
Respondents current working status		
Yes	3840	35.3
No	7031	64.7
Wealth status		
Poorest	3189	29.3
Poorer	1968	18.1
Middle	2067	19.0
Richer	1866	17.2
Richest	1781	16.4
Region		
Tigray	1032	9.5
Afar	875	8.0
Amhara	1037	9.5
Oromia	1313	12.1
Somalia	1045	9.6
Benishangul	739	6.8
SNNPR	1313	12.1
Gambela	764	7.1
Harari	655	6.0
Dire Dawa	808	7.4
Addis Ababa	1290	11.9

### Reproductive and obstetric characteristics of participants

The average number of living children was 2.2 per woman. Majority, 4538 (41.7%) of women had one to two living children. Only, 730 (6.7%) of the respondents had a history of terminated pregnancy. Regarding the desire to have children, 6245 (57.4%) reported that their husbands wanted to have children. More than one-third, 3888 (35.8%) of women want to have children after 2 years. The decision on contraceptive use mostly made by husband/partner alone 5912 (54.4%) and about 2512 (23.1%) made a joint decision when using a contraceptive method.

The majority of the participants (94.7%) had a good family planning knowledge. About two-fifths, 4307 (39.6%) of the women had visited a health facility in the last 12 months. Among these women visited the health facility in the last 12 months, 1533 (35.6%) had discussed with healthcare workers about family planning (FP), 1208 (28.0%) had counseled by healthcare workers regarding the side effects of contraceptives, and 1414 (32.8) had informed about the other family planning methods by healthcare workers (Table 2).

**Table 2:** Reproductive and obstetric characteristics of reproductive age women in Ethiopia, 2016 EDHS (weighted)

Variables	Number	Percent
Number of living children (parity)		
None	3246	29.9
1 to 2	4538	41.7
3 to 4	2373	21.8
5 and more	714	6.6
Husband wants to have an additional child?		
Yes	6245	57.4
No	4626	42.6
History of terminated pregnancy		
Yes	730	6.7
No	10,141	93.3
Fertility preference		
Wants within 2 years	2318	21.3
Wants after 2 years	3888	35.8
Wants no more children	2397	22.0
Undecided	2271	20.9
Decision maker for using contraception		
Mainly respondent	1990	18.3
Mainly husband/partner	5912	54.4
Jointly	2512	23.1
Others	457	4.2
Knowledge		
Good	10,291	94.7
Poor	580	5.3
Visited health facility in the last 12 months		
Yes	4307	39.6
No	6564	60.4
Discussed about FP with healthcare worker (n = 4307)		
Yes	1533	35.6
No	2774	64.4
Counseled by healthcare worker regarding the side effects (n = 4307)		
Yes	1208	28.0
No	3099	72.0
Information given regarding the other FP methods (n = 4307)		
Yes	1414	32.8
No	2893	67.2
Type of the last contraceptive used		
Pills	2458	22.6
IUD	493	4.6
Injectable	6113	56.2
Implant	1481	13.6
Condom	197	1.8
Traditional	129	1.2

### Prevalence of contraceptive discontinuation

**Fig. 1:**
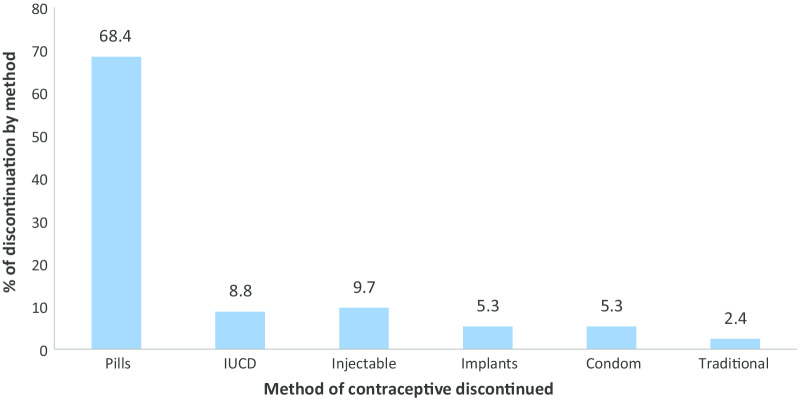
Contraceptive discontinuation by previous method used among reproductive age women in Ethiopia, 2016 EDHS

The prevalence of contraceptive discontinuation for all method among reproductive-age women was 32.2% (95% C.I 31.2, 33.1). The median duration of contraceptive use before discontinuation was 26 months with minimum 1 month and maximum 84 months. A higher proportion (66.8%) of contraceptive discontinuation was observed among mothers resided in rural areas as compared to urban dwellers (33.2%). The highest contraceptive discontinuation was reported among previous pills user 2394 (68.4%), and the lowest was observed among previous traditional methods users 84 (2.4%) (Fig. 1).

**Fig. 2:**
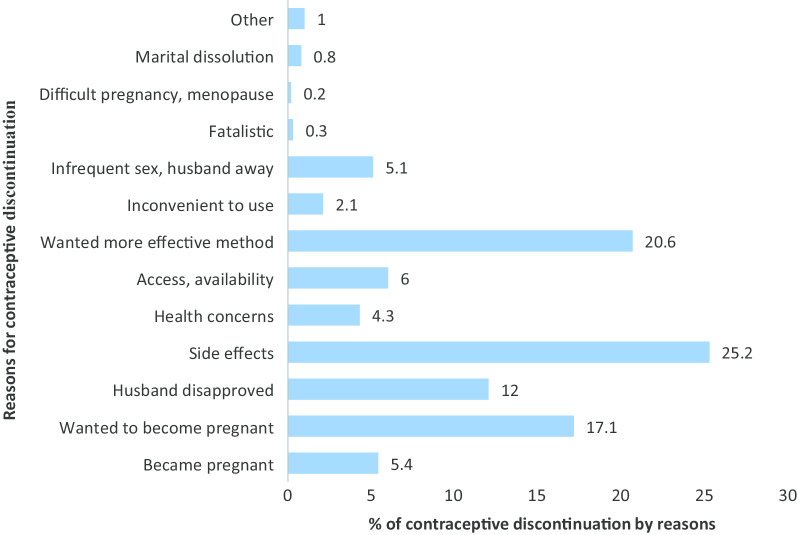
Reasons for contraceptive discontinuation among reproductive-aged women in Ethiopia, 2016 EDHS

The most common reasons given for discontinuing contraceptive method were side effect concern, wanted more an effective method, desired to become pregnant, husband/partner disapproval, unexpected conception, and other concerns. About one-fourth (25.2%) of women discontinued contraceptive use concerned about the side effects, (20.6%) wanted more an effective method, and (17.1%) for they wanted to get pregnancy (Fig. 2).

### Factors associated with contraceptive discontinuation

In multivariate logistic regressions analysis; place of residence, educational status of women, number of living children, husband desire for more children, decision making on contraceptive use, discussed about FP with healthcare worker and counseling about contraceptive side effects were significantly associated with discontinuation of contraceptives.

Women with rural residence were 1.94 times more likely to discontinue contraceptives (AOR = 1.94, 95% C.I 1.65, 2.28) compared to those women with the urban residence. Women with no formal education were 1.68 times more likely to discontinue contraceptives (AOR = 1.68, 95% C.I 1.30, 2.17). Women having no children (AOR = 1.95, 95% C.I 1.19, 3.58) were 1.95 times more likely to discontinue contraceptives compared to women with five and more children. In addition, women who reported that their husband wanted another child were nearly three times more likely to discontinue contraception (AOR = 2.57, 95% C.I 2.03, 3.26) than those not reporting this wish. Contraceptive discontinuation was less likely if a woman herself decided to use a contraceptive (AOR = 0.54, 95% C.I 0.38, 0.77) or its use was a joint decision within the couple (AOR = 0.38, 95% C.I 0.29, 0.48), rather than a decision made by others.

Women who didn’t discuss about FP with healthcare worker were 1.28 times more likely to discontinue contraceptive use (AOR = 1.28, 95% C.I 1.06, 1.54) compared to those women who discuss about FP with healthcare worker. Furthermore, women who didn’t get information about contraceptive side effects were about 2 times more likely to discontinue contraceptive use (AOR = 2.01, 95% C.I 1.59, 2.52) as compared to women who counseled by healthcare worker about contraceptive side effects (Table 3).

**Table 3:** Bivariate and multivariate analysis for contraceptive discontinuation among reproductive age women in Ethiopia, 2016 EDHS

Variables	Contraceptive discontinuation			
	COR (95% CI)	p-value	AOR (95% CI)	p-value
Marital status				
Married	0.88 (0.81, 1.06)	0.061	0.72 (0.57, 1.27)	0.361
Never married/divorced/widowed/separated	1		1	
Residence				
Urban	1		1	
Rural	1.36 (1.26, 1.47)	0.003	1.94 (1.65, 2.28)	0.001
Educational status				
No formal education	1.46 (1.27, 1.67)	0.001	1.68 (1.30, 2.17)	0.000
Primary school	0.71 (0.62, 0.82)	0.002	1.26 (0.89, 1.56)	0.210
Secondary school	0.68 (0.58, 0.80)	0.001	1.16 (0.92, 1.47)	0.301
College/University	1		1	
Respondents current working status				
Yes	1.46 (1.34, 1.58)	0.003	1.32 (0.91, 1.50)	0.191
No	1		1	
Number of living children (parity)				
None	0.82 (0.71, 0.97)	0.003	1.95 (1.19, 3.58)	0.005
1 to 2	1.89 (1.63, 2.20)	0.004	1.25 (0.96, 1.64)	0.101
3 to 4	0.66 (0.56, 0.78)	0.001	1.26 (0.94, 1.69)	0.122
5 and more	1		1	
History of terminated pregnancy				
Yes	2.76 (2.40, 3.16)	0.003	1.08 (0.89, 1.24)	0.435
No	1		1	
Fertility preference				
Wants within 2 years	0.42 (0.38, 0.47)	0.001	0.76 (0.47, 1.56)	0.082
Wants after 2 years	0.24 (0.22, 1.07)	0.091	0.83 (0.66, 1.35)	0.074
Wants no more children	0.12 (0.11, 0.14)	0.003	0.72 (0.51, 1.33)	0.083
Undecided	1		1	
Husband wants to have an additional child?				
Yes	3.35 (3.10, 3.6)	0.001	2.57 (2.03, 3.26)	0.000
No	1		1	
Decision maker for using contraception				
Mainly respondent	0.25 (0.21, 0.30)	0.001	0.54 (0.38, 0.77)	0.000
Mainly husband/partner	0.31 (0.26, 0.36)	0.001	0.69 (0.47, 1.76)	0.176
Jointly	0.33 (0.27, 0.39)	0.004	0.38 (0.29, 0.48)	0.001
Others	1		1	
Knowledge on contraceptives				
Good	2.79 (0.93, 3.44)	0.174	1.51 (0.49, 2.16)	0.301
Poor	1		1	
Discussed about FP with healthcare worker				
Yes	1		1	
No	1.71 (1.07, 1.91)	0.040	1.28 (1.06, 1.54)	0.009
Counseled by healthcare worker about side effects				
Yes	1		1	
No	1.61 (1.43, 1.81)	0.003	2.01 (1.59, 2.52)	0.001

## Discussion

This study was aimed to assess the prevalence and determinants of contraceptive discontinuation among reproductive-age women in Ethiopia using EDHS 2016 national survey. Accordingly, nearly one-third (32.2%) of the women who started an episode of contraceptive use preceding EDHS were discontinued within 12 months. The finding implies that the need of evaluating accessibility of different contraceptive methods and service quality regarding family planning. The finding also infers the need to provide comprehensive FP information which could help women to choose a suitable method that they can use for a longer period of time [20]. In addition, the finding of this study suggests the essentials of assessing the magnitude of unmet need for family planning as a nation. Evidence revealed that women who had ever used a modern method had discontinued its use yet still had an unmet need [8, 21]. Moreover, this discontinuation may be attributable to a reduced need for contraception, result from the fertility desire for another child by most women, poor satisfaction with the methods available or poor counseling on the management of side effects.

In this study, the prevalence of contraceptive discontinuation among reproductive-age women was 32.2% (95% C.I 31.2, 33.1). This finding was higher than DHS survey conducted from 34 countries, which reported discontinuation rate as 19% [11] and Myanmar DHS, which documented the prevalence of contraceptive discontinuation as 39.1% [22]. This finding was also higher than other studies done in Agarfa districts (25.5%), Southeast Ethiopia [17]. However, this finding is lower than study findings from 60 surveys in 25 countries with a discontinuation rate of 38% [7], analysis of Bangladesh DHS with 38.4% [18] and rural Bangladesh (36%) [23]. The reason for this variation may be due to differences in reproductive health service including information, counseling, and education that can increase comprehensive FP knowledge of women, availability and accessibility of different FP methods, and creating safe and convenient services for contraceptive users. In addition, the difference could be because of socio-demographic, beliefs, norms, and other cultural variations. The other reason could be due to the study period, sample size, study population, and sampling method.

In this study, high rate of contraceptive discontinuation was observed for pills. This finding is supported by other studies [18, 22, 24]. This indicated that women can take pills without proper counseling and information since pills are available at both public and private health facilities, pharmacies and drug shops. This suggests the indispensable of proper restrictions on marketing pills to unqualified individuals and the need for proper counseling on informed choice. On the other hand, the reason for the high rate of pills discontinuation could be due to its inconvenience to use that requires to be taken on a daily basis. Literature indicated that women choose pills to use as a temporary means of birth spacing; and they are more likely to discontinue [25].

This study found that concern about the potential side effects of contraceptives was the major reason for contraceptive discontinuation. This could imply that counseling and information services were inadequate regarding side effects. This result is similar with other studies carried out in different settings [18, 21, 22, 26, 27]. The other reported reason for contraceptive discontinuation was as of women wanted other more effective methods. This suggests the need for adequate and accurate information provision on timely initiation of contraceptives when women on method-switching.

In this study, factors that influence contraceptive discontinuation were identified. Women who lived in a rural resident were 1.94 times more likely to discontinue contraceptive compared with those living in an urban resident. This result is supported by other studies [5, 18, 22]. This could be explained by the fact that women from rural areas have poor access to reproductive health services and health care information including family planning service utilization [28]. Women with no formal education were 1.68 times more likely to discontinue contraceptives. This is also similar with other findings [18, 22, 26]. The reason might be due to the fact that women with no formal education have deprived autonomy to make decisions on reproductive health issues including when to use contraceptives [29].

As for fertility preference, women having no children were 1.95 times more likely to discontinue contraceptive methods compared with those women with five and more children. This finding is supported by other studies [17, 22, 26, 27]. The reason could be that women may need to have children to attain their desired number of children; thus they might prefer to discontinue contraceptives. Women who report their husbands desire for more children were 2.57 times more likely to discontinue contraceptives than those who perceive their husbands would not want more children. Similar studies have supported this finding from different settings [19, 22, 25, 27]. This implies that males are predominant decision-makers on contraception use in Ethiopia.

Furthermore, the odds of contraceptive discontinuation among women with self-decision when using a contraceptive method by themselves was 46% times less likely to discontinue and the odds of contraceptive discontinuation among women who made a joint decision with husband when using a contraceptive method were 62% times less likely to discontinue as compared to decision to contraceptive use made by others. This finding is supported by other studies conducted in Tanzania, Kenya, Nigeria [30–32]. This could be explained by the fact that women who supported by their husbands are less likely to discontinue contraception. Literature also suggests that couples’ joint decision making play an important role to use contraceptive method and its continuation [17].

This study also identified that women who didn’t discuss about FP with healthcare worker were 1.28 times more likely to discontinue contraceptive use. This implies that informed choice is an important principle in the provision of FP services; so that women can initiate other contraceptives immediately they switched the previous method. In addition, women who didn’t get information about contraceptive side effects were about 2 times more likely to discontinue contraceptive use than women who counseled. In line with this; evidence reported that poor counseling on side effects of contraceptive methods was a major cause of discontinuing of contraception [9, 19, 27]. The reason could be that women may frustrated while experiencing anything unusual related to contraceptive intake if they are not counseled appropriately about the side effects of contraceptives at the time dispensation; this would lead to a high probability contraceptive discontinuation.

### Strength and limitations of the study

This study used standardized data collection tools and nationally representative survey data with large sample size. Additionally, the study is representative of the whole regions of the country. However, respondents were likely to forget events that occurred for the past 5 years prior to the survey. Also, causal effects could not be measured because the study was based on a retrospective cross-sectional study. Some important determinant factors of contraceptive discontinuation were not examined due to high missing values in the data. Additionally, factors related with family, community, and some health system factors were not assessed, because this information was not fully available in the dataset.

## Conclusion

This study shows the prevalence of discontinuation of contraceptive method among reproductive-age women in Ethiopia was high which is close to EDHS report. Place of residence, educational status of women, number of living children, husband desire for more children, decision making on contraceptive use, discussion about FP with healthcare worker and counseling about contraceptive side effects were identified factors of discontinuation of contraceptive method. This finding suggests the need for quality counseling on side effects and availability of other contraceptive methods and partner involvement in decision making process with a collaborative effort of health care providers and other stakeholders. In addition, women empowerment should be promoted so that women are able to freely decide on when and how many children they wish to have. Moreover, special attention should be given to women with no formal education and from rural residents.

## Data Availability

The dataset of the EDHS is not available as a public domain survey dataset but can accessed with a request by registration on the MEASURE DHS website at: www.dhsprogram.com.

## Abbreviations

AOR: Adjusted odds ratio
CI: Confidence interval
COR: Crude odds ratio
EDHS: Ethiopian Demographic and Health Survey
FP: Family planning
MEASUREDHS: Monitoring and evaluation to assess and use results demographic and health surveys
SNNPR: Southern Nations, Nationalities, and Peoples’ Region
